# Quality-Score Refinement of SSU rRNA Gene Pyrosequencing Differs Across Gene Region for Environmental Samples

**DOI:** 10.1007/s00248-012-0043-9

**Published:** 2012-04-05

**Authors:** Kara Bowen De León, Bradley D. Ramsay, Matthew W. Fields

**Affiliations:** 1 Department of Microbiology, Center for Biofilm Engineering, Montana State University, 366 EPS Building, Bozeman, MT 59717 USA; 2 Thermal Biology Institute, Montana State University, Bozeman, MT USA; 3 ENIGMA, http://enigma.lbl.gov/

## Abstract

Due to potential sequencing errors in pyrosequencing data, species richness and diversity indices of microbial systems can be miscalculated. The “traditional” sequence refinement method is not sufficient to account for overestimations (e.g., length, primer errors, ambiguous nucleotides). Recent in silico and single-organism studies have revealed the importance of sequence quality scores in the estimation of ecological indices; however, this is the first study to compare quality-score stringencies across four regions of the SSU rRNA gene sequence (V1V2, V3, V4, and V6) with actual environmental samples compared directly to corresponding clone libraries produced from the same primer sets. The nucleic acid sequences determined via pyrosequencing were subjected to varying quality-score cutoffs that ranged from 25 to 32, and at each quality-score cutoff, either 10 or 15 % of the nucleotides were allowed to be below the cutoff. When species richness estimates were compared for the tested samples, the cutoff values of Q27_15%_, Q30_10%_, and Q32_15%_ for V1V2, V4, and V6, respectively, estimated similar values as obtained with clone libraries and Sanger sequencing. The most stringent Q tested (Q32_10%_) was not enough to account for species richness inflation of the V3 region pyrosequence data. Results indicated that quality-score assessment greatly improved estimates of ecological indices for environmental samples (species richness and α-diversity) and that the effect of quality-score filtering was region-dependent.

**Electronic supplementary material:**

The online version of this article (doi:10.1007/s00248-012-0043-9) contains supplementary material, which is available to authorized users.

## Introduction

Pyrosequencing [1] of small subunit (SSU) rRNA gene amplicons has permitted sampling at an unprecedented depth, providing orders of magnitude more sequence information than Sanger sequencing of clone libraries, and deeper coverage has typically estimated more diversity than was previously recognized [2]. However, intrinsic errors during pyrosequencing may overestimate species diversity by as much as an order of magnitude [3, 4]. Methods to alleviate inflated species richness estimates include quality-score analysis and modifications to alignment and/or clustering methods [3, 5, 6]. Both techniques can result in lower estimations of α-diversity; however, validation is needed with actual environmental samples.

Quality-score analysis is a quick method to remove error-prone sequences from the fasta files alleviating compatibility issues with downstream applications. Phred quality scores (Q) range from 0 to 40 and are typically assigned by the sequence determination software based upon confidence in the base call. Kunin et al. [3] tested the applicability of quality-based end trimming to alleviate artificial inflation of species richness estimates using a single-organism culture and recommended trimming each sequence until all nucleotides have a Q ≥27 for FLX reads. In a subsequent study, Kunin and Hugenholtz [6] recommended quality-based refinement without trimming but with thresholds that allowed a certain percentage of bases to have a Q <27 via PyroTagger (noting that >80 % of reads may be removed at this stringency threshold). While this method has been validated with a single-species laboratory culture for the V1V2 and V8 SSU rRNA gene regions, it has not been evaluated with an actual environmental sample, for other regions of the SSU gene, or for 454 titanium reads. In this study, we used a water sample from the Hanford 100H site in the Hanford Nuclear Reservation to compare titanium pyrosequencing at varying Q cutoffs to a large clone library for the V1V2 and V3 regions of the bacterial SSU rRNA gene. Furthermore, we used a thermoalkaline spring slurry sample from Yellowstone National Park to compare Q cutoff analyses of the V4 and V6 regions to large clone libraries. The results verified that Q assessment should be used for ecological characterization of real environmental samples, but showed that the effect of Q filtering was region-dependent unlike previous studies that have tested the predictions with monocultures.

## Methods

### Sample Collection and Preparation

A water/soil slurry sample from a hot spring in the Heart Lake Geyser Basin of Yellowstone National Park (44.29068 N, 110.50983 W) was collected in a 50-ml conical vial and stored at −80°C. After centrifugation at 6,000×*g* for 20 min, 4.6 g of the pellet was used for extraction. Groundwater (1 L) from well 699-96-41 of the 100H site in the Hanford Nuclear Reservation was filtered, and the filters were stored at −80°C (bottle top vacuum filter, 0.22-μm-pore PES membrane, Corning Inc., Corning, NY, USA). Approximately one half of the filter was rinsed with 100 mM phosphate buffer (pH 7) and vortexed for 30 s, settled, and then repeated. Sterile sand was added to the biomass-containing buffer and ground as described below.

### DNA Extraction and Sequencing

Samples were suspended in MO BIO PowerMax™ Soil DNA Isolation Kit PowerBead Solution, and cells were disrupted using two cycles of freeze–thaw and grinding with a mortar and pestle, as previously described [7] (MO BIO Laboratories Inc., Carlsbad, CA, USA). DNA was extracted following the protocol of the MO BIO Kit mentioned above. The DNA was cleaned and concentrated with the Wizard® SV Gel and PCR Clean-Up System (Promega Corporation, Madison, WI, USA) according to the manufacturer's protocol.

SSU rRNA gene sequences were amplified via 25 cycles of PCR (10–12 ng DNA/reaction) with barcoded universal bacterial primers FD1 (5′-ctcgcgtgtcAGAGTTTGATCCTGGCTCAG-3′) and 529R (5′-ctcgcgtgtcCGCGGCTGCTGGCAC-3′) for the V1V2 and V3 regions (Hanford sample) and 530F (5′-tagtgtagatGTGCCAGCMGCNGCGG-3′) and 1100R (5′-tagtgtagatGGGTTNCGNTCGTTR-3′) for the V4 and V6 regions (Yellowstone sample) under the conditions described previously [8]. PCR products were excised from a 0.8 % agarose gel and pooled using an Ultrafree®-DA gel extraction column (Millipore Corporation, Bedford, MA, USA). The gel extract was cleaned and concentrated using the Wizard® SV Gel and PCR Clean-Up System, and dsDNA was quantified with a Qubit fluorometer (Invitrogen, Carlsbad, CA, USA). Adaptors for 454 sequencing were ligated to the amplicons and were pyrosequenced on a 454 GS-FLX Titanium™ (454 Life Sciences, Branford, CT, USA) at SeqWright, Incorporated (Houston, TX, USA). Clone libraries were constructed with the same primers listed above and purified as previously described [8], with modifications in vector (pCR®4-TOPO®, Invitrogen) and sequencing primer (M13F(−20) (5′-GTAAAACGACGGCCAG-3′) sequence provided with vector). Clonal sequences were determined at Functional Biosciences Incorporated via capillary sequence determination (Madison, WI, USA).

### Sequence Refinement

Sequences were trimmed to one standard deviation below the mean (removed if shorter), subjected to varying Q cutoffs (25, 27, 30, and 32) allowing either 10 or 15 % of the nucleotides to be below the cutoff, and removed if primer errors or ambiguous nucleotides were observed. An in-house python script was used for data management and analyses. The python scripts with example output files and a readme file have been uploaded to https://bitbucket.org/kbdeleon/seqrefinement/ and are publicly available. The upfront analysis of our seqrefinement provides a fasta file that can be used for many typical downstream analyses, such as the ChimeraSlayer and RDP pipeline, as described in this study. Chimeras were removed using ChimeraSlayer [9]. The RDP Pyrosequencing Pipeline was used to align sequences, complete-linkage cluster at 97 % similarity, and generate rarefaction curves. Clone library sequences were extracted from chromatograms and vector sequences removed in eBioX (v1.5.1; http://www.ebioinformatics.org/ebiox/). Clonal sequences were subjected to the same refinement conditions as the sequences determined via pyrosequencing except for Q analyses.

## Results and Discussion

Pyrosequencing and clone library sequence sets were generated for the V1V2, V3, V4, and V6 regions of the SSU rRNA gene sequence using the same barcoded primers for both methods to alleviate possible primer biases. The sequences were subjected to the traditional method of sequence refinement including removal of sequences shorter than one standard deviation from the mean length (minimum length, 246 nt for V1V2 and V3 and 253 nt for V4 and V6) and those that contained primer errors or ambiguous nucleotides. The mean average Q varied from 28.8 to 30.6 and increased to 32.3–34.4 upon trimming to the minimum length (Online resource 1). The guidelines proposed by Kunin et al. [3] of quality-based end trimming or by Kunin and Hugenholtz [6] of trimming but allowing 3 % of bases to be <Q27 were considered but removed >99 or >93 % of the environmental sequence sets, respectively (Online resource 1). However, PyroTagger (http://pyrotagger.jgi-psf.org/cgi-bin/index.pl), the program resulting from Kunin and Hugenholtz [6], recommends the allowance of 10 to 15 % of bases with <Q27 for titanium pyrosequencing. Our study directly evaluated the impact of Q cutoff on species richness and diversity estimates by comparing clone library and pyrosequencing results for the same sample with the same DNA, same PCR primers, and same barcodes. The sequences were subjected to Q25, 27, 30, and 32 that allowed 10 or 15 % to be below the Q threshold (hereafter designated as a subscript of the Q) (Tables 1 and 2). These parameters resulted in 68 to 95 % removal of sequences after refinement and quality check depending on stringency and SSU rRNA gene region.

**Table 1:** Sequence removal during refinement of V1V2 and V3 SSU rRNA gene region sequences (32,517 raw sequences) and clone libraries

	Full length–no Q	Trimmed–no Q	Q25_15%_	Q25_10%_	Q27_15%_	Q27_10%_	Q30_15%_	Q30_10%_	Q32_15%_	Q32_10%_	Clone library^a^
Length, <246 nt	5,957	5,957	5,957	5,957	5,957	5,957	5,957	5,957	5,957	5,957	0
Quality	–	–	10,036	15,542	13,399	18,835	17,787	22,280	20,456	24,039	–
Ns	3,285	761	143	49	71	21	29	4	10	1	23
Primer errors	413	467	203	126	154	95	101	46	72	29	70
V1V2 does not meet NAST reqs^b^	478	1,068	215	127	158	77	90	40	63	29	34
V1V2 chimeric	356	344	188	115	145	77	87	31	56	15	14
V3 does not meet NAST reqs^b^	57	66	42	27	32	18	20	15	16	10	0
V3 chimeric	1,259	2,710	1,984	1,409	1,651	1,020	1,140	586	824	332	66
Sequences remaining	20,712	21,144	13,749	9,165	10,950	6,417	7,306	3,558	5,063	2,105	1,331
V1V2 remaining	10,478	11,224	6,377	3,673	4,723	2,247	2,707	1,009	1,600	484	677
V3 remaining	10,234	9,920	7,372	5,492	6,227	4,170	4,599	2,549	3,463	1,621	654
% sequences removed	36.3 %	35.0 %	57.7 %	71.8 %	66.3 %	80.3 %	77.5 %	89.1 %	84.4 %	93.5 %	–

^a^The V1–V3 region was sequenced together, cut into V1V2 and V3, and treated as two separate datasets. Numbers shown are totals of the two datasets

^b^NAST parameters set to default except the minimum length which was set to 200 nt

**Table 2:** Sequence removal during refinement of V4 and V6 SSU rRNA gene region sequences (18,628 raw sequences) and clone libraries

	Full length–no Q	Trimmed–no Q	Q25_15%_	Q25_10%_	Q27_15%_	Q27_10%_	Q30_15%_	Q30_10%_	Q32_15%_	Q32_10%_	Clone library
Length, <253 nt^1^	3,530	3,530	3,530	3,530	3,530	3,530	3,530	3,530	3,530	3,530	16
Quality	–	–	6,866	9,772	8,850	11,570	11,127	13,278	12,530	14,090	–
Ns	2,104	467	54	23	27	7	8	1	5	0	0
Primer errors	480	538	264	167	201	120	133	64	85	33	–
V4 does not meet NAST reqs^a^	356	431	215	132	163	85	100	46	68	28	18
V4 chimeric	265	322	200	129	156	86	104	43	72	19	11
V6 does not meet NAST reqs^a^	79	93	50	24	31	13	17	7	12	6	4
V6 chimeric	353	231	56	18	28	9	12	4	8	2	12
Sequences remaining	11,461	13,016	7,393	4,833	5,642	3,208	3,597	1,655	2,318	920	1,113
V4 remaining	5,819	6,639	4,623	3,452	3,803	2,458	2,680	1,398	1,812	814	695
V6 remaining	5,642	6,377	2,770	1,381	1,839	750	917	257	506	106	418
% sequences removed	38.5 %	30.1 %	60.3 %	74.1 %	69.7 %	82.8 %	80.7 %	91.1 %	87.6 %	95.1 %	–

^a^NAST parameters set to default except minimum length was set to 200 nt

**Figure 1:**
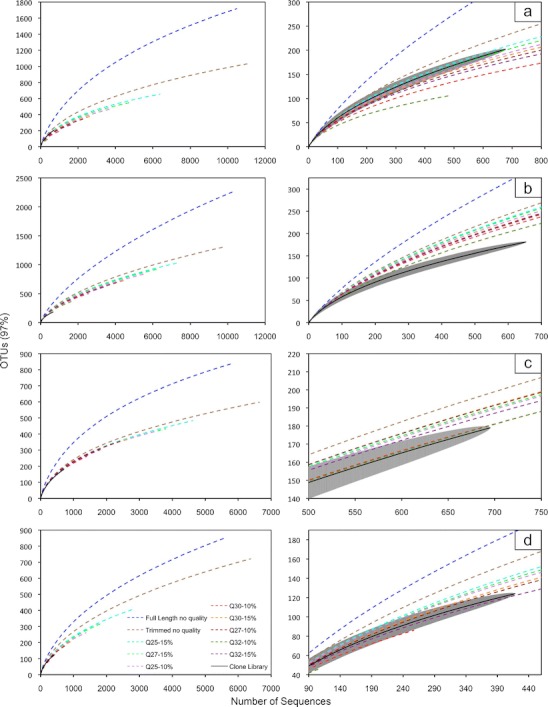
Species richness estimates for the V1V2 (**a**), V3 (**b**), V4 (**c**), and V6 (**d**) SSU rRNA gene regions. Full and enlarged rarefaction curves are displayed for each region of the SSU rRNA gene. Operational taxonomic units (OTUs) are clustered at 97 % similarity. The *wide gray line* in the enlarged rarefaction curves represents 95 % confidence intervals for the clone library species richness predictions

A comparison of species richness via rarefaction curves demonstrated a dependence of species estimates on trimming and quality checking (Fig. 1). In all cases, species richness was significantly higher for non-trimmed sequences and trimmed sequences without Q analysis. The corresponding clone library was used as a guide to determine the best Q cutoff for each SSU rRNA gene region. The data suggested that a Q cutoff is not universal across different regions of SSU rRNA gene sequences. Q27_15%_ yielded a similar species richness projection to the clone library for the V1V2 region, corresponding to the single-species findings of Kunin et al. [3]. For the V3 region, the most stringent Q cutoff of 32_10%_ was not sufficient to reduce the species richness estimates to the point predicted by the clone library. Q30_10%_ and Q32_10%_ resulted in similar estimates as the trimmed clone library for the V4 region; however, Q32_10%_ is on the same trajectory as Q30_10%_, but with less sequences due to the increased quality stringency. For the V6 region, Q32_15%_ resulted in similar species richness estimates as the clone library. It is important to note for the V1V2 and V6 regions, the Qs tested could be too stringent and resulted in underestimated species richness compared to the clone library. Thus, attempting to use a universal Q cutoff for all regions of the SSU rRNA gene sequence is not feasible and could lead to over- or underestimation of the species richness depending on the SSU rRNA gene region.

**Figure 2:**
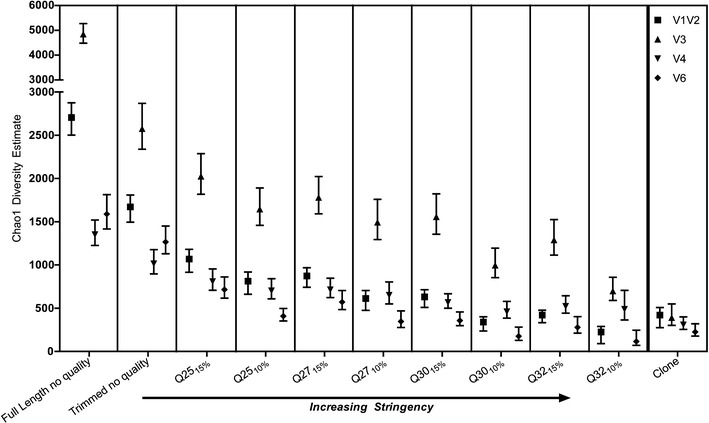
Chao1 diversity estimates for the same samples at different Q filtering compared to the respective clonal library. *Error bars* denote 95 % confidence intervals

Chao1 diversity estimates further stressed the importance of quality-filtering pyrosequencing data after “traditional” refinement (Fig. 2). Full-length sequences without quality check resulted in a Chao1 diversity estimates up to 12-fold higher than that of the corresponding clone library while traditional refinement with the addition of trimming to the minimum length still resulted in almost sevenfold overestimations of diversity (Fig. 2). As expected, the Chao1 decreased gradually as the stringency of the Q cutoff increased. The Chao1 predictions support the Q cutoffs suggested by the rarefaction curves and further stress the need for Q analysis.

Because Chao1 can be influenced by sample size [10–12], a Q cutoff for each SSU rRNA gene region cannot be recommended solely on Chao1, and random subset generation can help alleviate the influence of sample size on Chao1. However, when comparing pyrosequencing to clone libraries, random subsets are not feasible due to the size limitations of clone libraries, and this would greatly diminish the added resolution of species diversity provided by the large sample sizes of pyrosequencing. Nevertheless, when Chao1 diversity estimates across samples were compared with and without Q refinement, the results demonstrated the necessity of further sequence refinement and provided a validated, threshold Q stringency.

**Figure 3:**
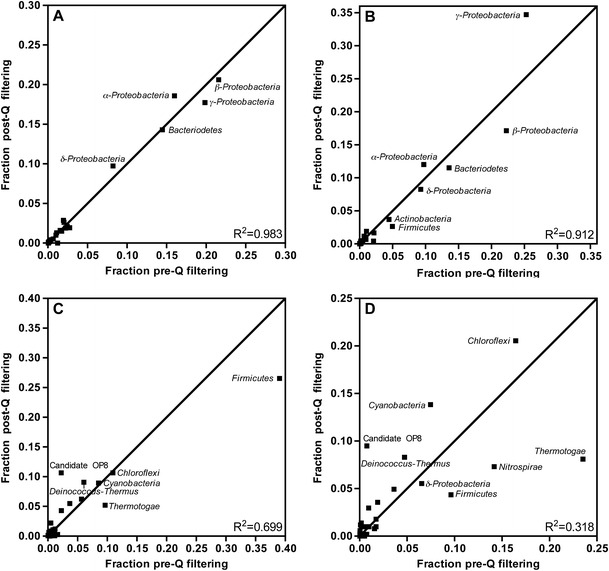
Phylogenetic comparison pre- and post-quality filtering. The phylum (class for *Proteobacteria*) distribution was compared for each region of the SSU rRNA gene at the Q suggested by the rarefaction curves in Fig. 1 (Q27_15%_ for V1V2 (**a**), Q32_10%_ for V3 (**b**), Q30_10%_ for V4 (**c**), and Q32_15%_ for V6 (**d**)). The coordinates for each taxon correspond to the abundance by fraction of unfiltered sequences (*x*-axis) and fraction of filtered high-quality sequences (*y*-axis). The scale differs across graphs to maximize point separation. Taxa along the line of *y* = *x* did not show a shift in percent abundance during filtering while those left and above the line represent phylogenetic groups that shifted to higher abundance post-filtering, and those right and below the line had a lower abundance post-filtering. Linear regression analysis to the line *y* = *x* yielded *R* ^2^ values that indicate how well each region fits the assumption that the sequences removed were not phylogenetically biased

One concern of using Q to refine pyrosequencing samples is that the sequences removed from the dataset are biased towards a certain phylogenetic group, thus artificially skewing the distribution towards or away from certain organisms (e.g., sequences with conserved homopolymers). We compared the phylum distribution (class for *Proteobacteria)* for each SSU rRNA gene region at the Q cutoffs suggested above (V1V2: Q27_15%_, V3: Q32_10%_, V4: Q30_10%_, V6: Q32_15%_) both before and after Q filtering (Fig. 3). Regression analysis with the predicted values of *y* = *x* (no difference in phylogenetic distribution pre- and post-quality filtering) was used to compare how well the data fit the assumption of no bias in sequence removal. The V1V2 region data fit the predicted values quite well (*R* ^2^ = 0.98) and thus was not biased in sequence removal for the phyla present in the sampled diversity. As expected, distributions could change at the resolution of genus (Online resource 2). For example, genera within *Bacteriodetes* remained at similar distributions (*R* ^2^ = 0.91), but some genera distributions within the β-*Proteobacteria* were altered (*R* ^2^ = 0.75). The results highlighted the importance of filtering pyrosequence data, particularly for α-diversity.

The V3 region may slightly skew the percent abundance towards γ-*Proteobacteria* and away from β-*Proteobacteria* (*R* ^2^ = 0.91). For the V4 region, all candidate OP8 sequences were high quality, so the percent abundance increased post-filtering, and *Firmicutes* and *Thermotogae* became less dominant post-filtering (*R* ^2^ = 0.70). The V6 region was quite skewed in Q-based sequence removal (*R* ^2^ = 0.32). In this region, *Thermotogae*, *Nitrospirae*, and *Firmicutes* decreased in percent abundance while *Chloroflexi*, *Cyanobacteria*, *Deinococcus*-*Thermus*, and candidate OP8 increased in abundance. Previous studies have raised issues with the use of the V6 region for microbial community analyses [13, 14], and our presented data corroborate these findings. It is interesting to note that this region of the SSU rRNA gene is known to have few homopolymers [15], and the analysis of sequences from the affected groups did not indicate a trend in the presence of homopolymers as a cause for removal (data not shown). There are likely other characteristics of this especially hypervariable region that could contribute to the observed bias. Nevertheless, extra caution must be taken when attempting to use this region of the SSU rRNA gene for OTU distribution predictions.

**Figure 4:**
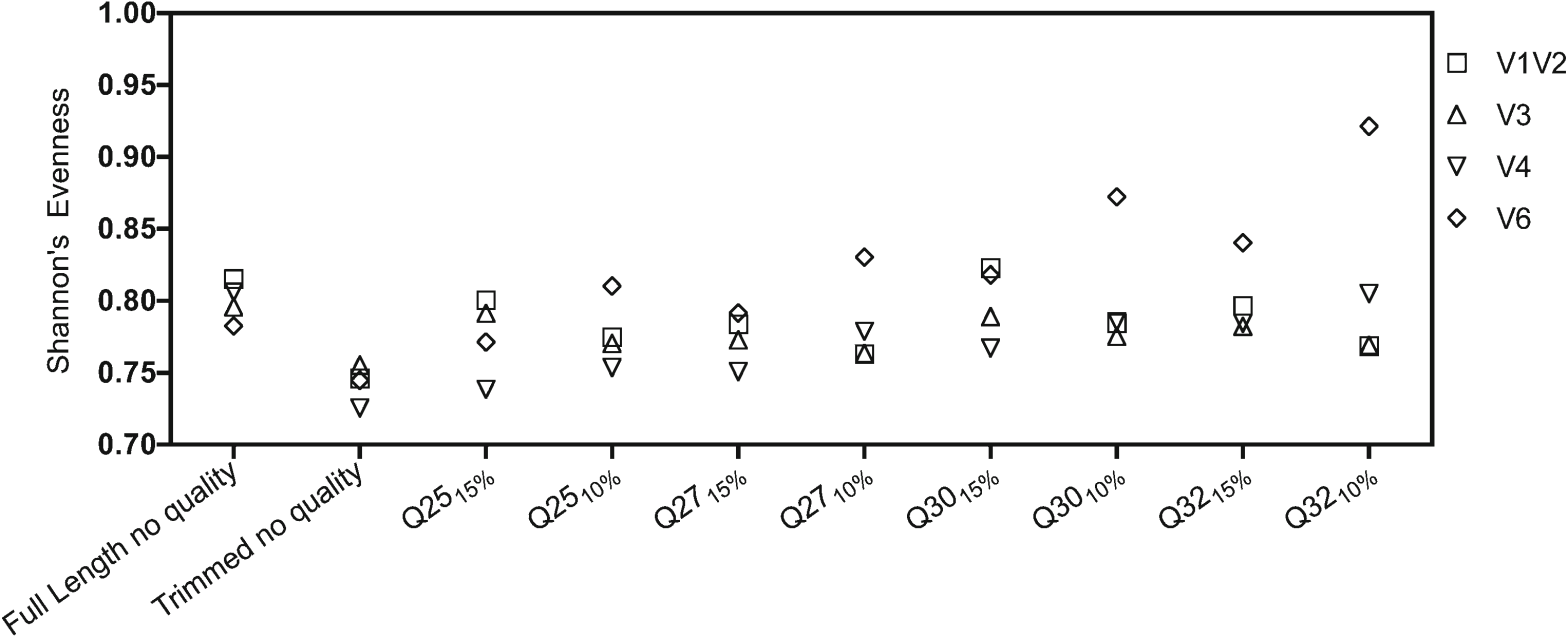
Shannon's evenness for each respective library with increasing stringency of Q filtering

An increase in quality stringency yielded a slight increase in species evenness (Fig. 4). An increase in species evenness can be due to the removal of low-abundance artifacts and/or a reduction in size of the largest clusters. Many errors are likely in the singleton and doubleton clusters, yet the clusters of dominant organisms likely contain a larger percentage of the erroneous sequences purely based on numerical dominance. The largest increase in evenness was observed when the V6 region was Q filtered, and this result coincides with the observation in Fig. 3 that the V6 region was more susceptible to phylogenetic bias.

**Figure 5:**
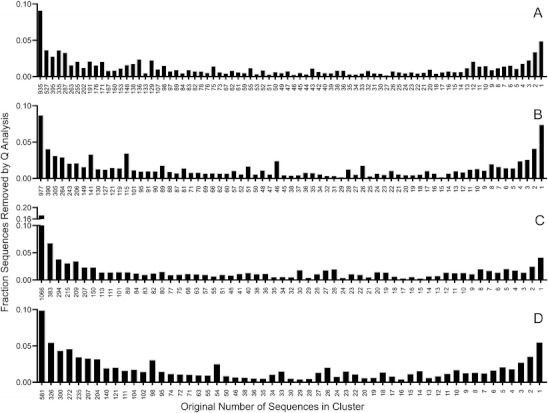
Fraction of total sequences removed from clusters which have been trimmed but not quality filtered during Q analysis. Sequences were clustered pre-quality checking, and the cluster in which sequences were removed during quality checking was monitored for the V1V2 (**a**), V3 (**b**), V4 (**c**), and V6 (**d**) SSU rRNA gene regions. The Q parameter was Q27_15%_ for V1V2, Q32_10%_ for V3, Q30_10%_ for V4, and Q32_15%_ for V6. The majority of sequences were removed from the largest and smallest clusters

To ensure that low-quality sequences were being removed from clusters most likely to contain erroneous sequences, we examined which clusters contributed most to percent sequence removal during Q analysis at the cutoff determined for each region (Fig. 5). Each resulted in a parabolic curve in which the majority of sequences were removed from the largest and smallest clusters and less from the mid-sized clusters. Thus, low-quality sequences were being removed from clusters with the highest likelihood of containing sequencing errors.

In an attempt to explain the differences in Q stringency requirements for each SSU rRNA gene region, homopolymer incidence and length were examined (Table 3). The GS20 quality score has been previously used as a measure of confidence that a homopolymer length is correct at a given position [15]. Furthermore, poly-A/T homopolymers tend to be more problematic [16]. The V3 region had the highest incidence of long poly-T homopolymers and required the most stringent Q cutoff to alleviate species richness inflation (Q32_10%_). In addition to conserved and semi-conserved regions, a variable stem-loop (P17-1) is present in the bacterial V3 region [17], and this additional heterogeneity may contribute to increased sequencing error. The V1V2 and V4 had a higher prevalence of poly-G homopolymers with the V4 region having one sequence with a ten-nucleotide poly-G stretch. The V6 region had a lower incidence of long homopolymers, as previously reported [15], but tended to have poly-C stretches when homopolymers occurred. Such long homopolymers also occurred in the clone library sequences and thus are not purely artifactual. Our results suggested that homopolymer stretches contribute to the observed pyrosequencing biases, but did not solely explain the region-dependent differences.

**Table 3:** Homopolymer occurrence in pyrosequencing and clone libraries

VIV2	Pyrosequencing						Clone library					
	4mers	5mers	6mers	7mers	8mers	>8mers	4mers	5mers	6mers	7mers	8mers	>8mers
A	2,364	796	148	4	1	0	93	35	0	0	0	0
T	1,160	1,051	210	26	0	0	58	71	15	0	0	0
G	9,178	10,843	629	77	17	0	537	849	18	6	0	0
C	2,709	325	85	24	0	0	137	13	3	0	0	0
V3												
A	6,820	552	20	2	0	0	570	4	1	0	0	0
T	1,546	487	295	182	1	0	110	24	14	10	0	0
G	1,473	787	621	93	2	0	102	54	28	1	0	0
C	8,438	3,596	403	28	0	0	620	256	33	0	0	0
V4												
A	3,064	188	32	1	0	0	137	2	0	0	0	0
T	470	20	0	0	0	0	46	2	0	0	0	0
G	6,393	3,349	450	37	23	5^a^	652	387	31	0	4	0
C	813	609	45	0	0	0	66	80	0	0	0	0
V6												
A	579	58	8	0	0	0	43	3	1	0	0	0
T	1,678	272	2	0	0	0	137	9	0	0	0	0
G	2,599	831	8	1	0	0	278	4	0	0	0	0
C	8,849	6,551	433	16	5	1^b^	703	392	4	0	0	0

^a^Four 9mers and one 10mer

^b^One 9mer

Pyrosequencing is quickly replacing capillary sequencing of clone libraries as the standard technique for molecular and ecological studies of microbial communities due to breadth, depth, and cost. However, only recently have the potential impacts of sequence quality (e.g., error rates) been considered (referenced above) with respect to ecological estimates for community composition and structure. While other methods of buffering the data against erroneous sequences through different alignment and clustering methods can be used [5], quality checking is a complementary method that can quickly remove error-prone sequences using the quality score file that commonly accompanies flowgram processing and output. Previous clone library analyses have shown that similarity values below 0.995 are not due to sequencing errors (95 % CI) with capillary-based sequence determination [18], and clone library sequences were thus used for comparison to pyrotagged sequence sets. In addition, recent work has shown that FLX sequence determination has comparable error rates to capillary sequencing when Q averages 24 to 27 [5]. Thus, we performed a direct comparison of clone libraries to pyrosequence libraries from two environmental samples for four regions of the SSU rRNA gene sequence in order to validate ecological estimations of sampled diversity from two different environments. It should be noted that clone libraries could underestimate sampled diversity due to limited sampling size; however, the clone libraries for this study were large (418 to 694 clones/gene region) and were used as a conservative estimate for which to compare pyrotag data. While this method is conservative, it provides a baseline validation of pyrotag sequencing for microbial communities. We do not provide the predictions from this comparison as an absolute value, but rather as a means to establish lower and upper thresholds compared to previous techniques.

This is the first study to test and validate the effects of quality-based refinement on real sampled diversity, and our results further stress the importance of Q for pyrosequence data filtering in a region-dependent manner for accurate estimations of species richness. With our tested samples, we observed that the quality scores that best fit the V1V2, V4, and V6 regions were Q27_15%_, Q30_10%_, and Q32_15%_, respectively, and the most stringent Q tested (Q32_10%_,) was not enough to account for species richness inflation of the V3 region. It is possible that these stringencies may be sample or sample-type specific, but the results from the different environmental samples that tested four different regions of the SSU rRNA gene sequence all showed the necessity of quality-score refinement. The results suggested that the region dependence of parameters should be tested and considered during experimental design (e.g., gene region, sample type) when using pyrotagged community analyses. Accurate α-diversity estimations will become increasingly important in light of environmental meta-omics approaches, as well as accurate predictions of β- and γ-diversity for providing insight into structure–function relationships.

## Electronic supplementary material

Below is the link to the electronic supplementary material.

Online Resource 1 Application of previously published Q refinement methods to real sample data (PDF 21.5 kb)

Online Resource 2 Genus-level phylogenetic comparison of the V1V2 region pre- and post-quality filtering. Genera from *Bacteriodetes* (**a**) and *β-proteobacteria* (**b**) are displayed as examples from phyla that showed minimal differences in relative abundance pre- and post-quality filtering. The coordinates for each genus correspond to the abundance by fraction of unfiltered sequences (*x*-axis) and fraction of filtered high-quality sequences (*y*-axis). Linear regression analysis to the line *y* = *x* yielded *R* ^2^ values that indicate how well genera from each phylum fit the assumption that the sequences removed were not biased at the genus level (PDF 1,328 kb)
