# Supplementary material for: Quality-Score Refinement of SSU rRNA Gene Pyrosequencing Differs Across Gene Region for Environmental Samples
Source: Microb Ecol. 2012 Apr 5;64(2):499–508. doi: 10.1007/s00248-012-0043-9 (PMC3391548; doi:10.1007/s00248-012-0043-9)
Supplement: Supplementary file 1 — Application of previously published Q refinement methods to real sample data (PDF 21.5 kb) [file 248_2012_43_MOESM1_ESM.pdf]

Online Resource 1. Application of previously published Q refinement methods to real sample data.

**Full Length**	**V1V2**	**V3**	**V4**	**V6**
Sequences post-traditional[a] refinement	10478	10234	5819	5642
Mean average quality score	30.0	30.6	29.2	28.8
Sequences remaining after Q27 refinement	0	0	0	0
Sequences remaining after Q27 refinement allowing 3%	0	8	0	0
**Trimmed**				
Sequences post-traditional[a] refinement	11224	9920	6639	6377
Mean average quality score	33.1	34.4	33.8	32.3
Sequences remaining after Q27 refinement	6	78	27	1
Sequences remaining after Q27 refinement allowing 3%	163	694	333	41
**Quality-Based End-Trimming**				
Average length after end-trimming to Q27	85.7	90.2	76	61.1
Sequences remaining > minimum length	5	78	24	1

[a]Traditional refinement includes removal of sequences with length <1sd from mean length, primers errors, and Ns
