# Supplementary figures and images for: Quality-Score Refinement of SSU rRNA Gene Pyrosequencing Differs Across Gene Region for Environmental Samples
Source: Microb Ecol. 2012 Apr 5;64(2):499–508. doi: 10.1007/s00248-012-0043-9 (PMC3391548; doi:10.1007/s00248-012-0043-9)

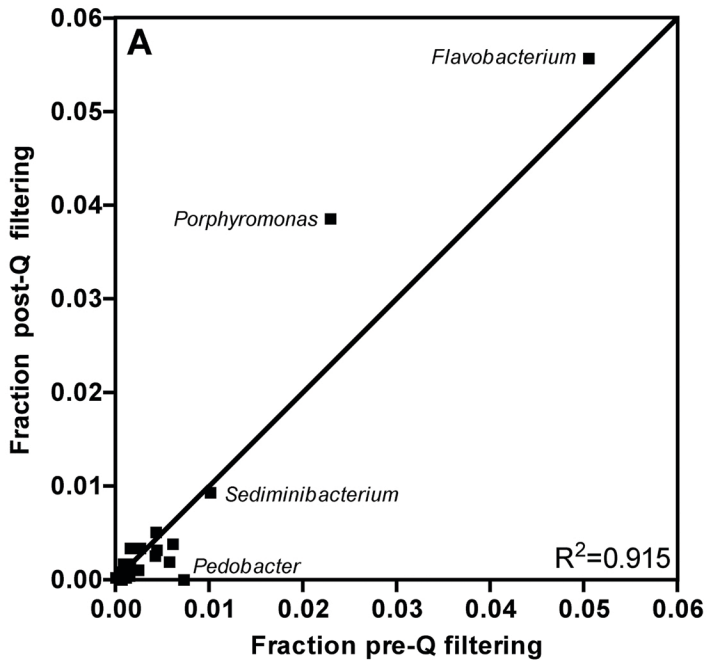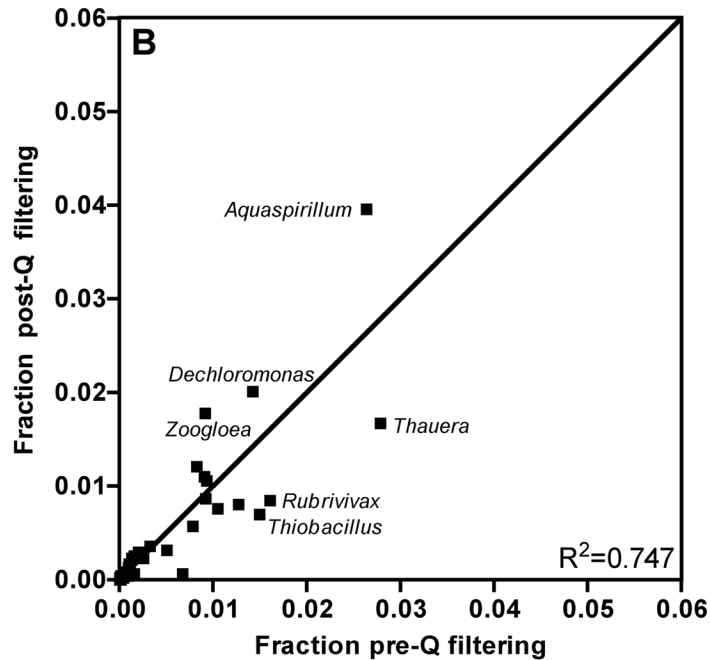

Supplement: Supplementary file 2 — Genus-level phylogenetic comparison of the V1V2 region pre- and post-quality filtering. Genera from Bacteriodetes (a) and β-proteobacteria (b) are displayed as examples from phyla that showed minimal differences in relative abundance pre- and post-quality filtering. The coordinates for each genus correspond to the abundance by fraction of unfiltered sequences (x-axis) and fraction of filtered high-quality sequences (y-axis). Linear regression analysis to the line y = x yielded R 2 values that indicate how well genera from each phylum fit the assumption that the sequences removed were not biased at the genus level (PDF 1,328 kb) [file 248_2012_43_MOESM2_ESM.pdf]
